# The Effects of Positive Airway Pressure Ventilation during Cardiopulmonary Bypass on Pulmonary Function Following Open Heart Surgery

**DOI:** 10.5812/cardiovascmed.8129

**Published:** 2013-05-20

**Authors:** Mostafa Alavi, Behshid Pakrooh, Yalda Mirmesdagh, Hooman Bakhshandeh., Touraj Babaee, Saeid Hosseini, Faranak Kargar

**Affiliations:** 1 Rajaie Cardiovascular Medical and Research Center, Tehran University of Medical Sciences, Tehran, IR Iran; 2 Heart Valve Disease Research Center, Rajaie Cardiovascular Medical and Research Center, Tehran University of medical sciences, Tehran, IR Iran

**Keywords:** Continuous Positive Airway Pressure, Coronary Artery Bypass Grafting, Cardiopulmonary Bypass

## Abstract

**Background:**

Intrapulmonary shunt as a result of atelectasis following cardiac surgeries is an important and common postoperative complication that results into pulmonary dysfunction typically lasting more than a week following surgery. Different methods have been provided to prevent these complications.

**Objectives:**

In order to prevent postoperative pulmonary complications, investigation of the effectiveness of continuous positive airway pressure (CPAP) and intermittent mandatory ventilation (IMV) during cardiopulmonary bypass (CPB) in patients undergoing coronary artery bypass grafting (CABG).

**Materials and Methods:**

In this prospective interventional study, 300 patients, candidate for elective CABG (On-Pump), were randomly allocated to 3 groups: A, B, C. Group A (CPAP) patients received CPAP at 10 cm H_2_O during CPB. Group B (IMV) patients received IMV with a tidal volume of 2 cc/kg and respiratory rate of 15/min and group C (control) patients did not receive any type of ventilation during CPB. Other procedures were similar between groups. Arterial blood samples were taken at 8 moments and arterial blood gas (ABG) analysis were compared between groups. Chest x-rays after CABG were also evaluated with respect to atelectasis.

**Results:**

The demographic data were similar in between three groups. Graft number, pump time and preoperative ABGs were not significantly different. Postoperative PaO_2_ were significantly higher in the CPAP and IMV groups and (A-a) DO2 were significantly lower in these two groups, compared to the control group.

**Conclusions:**

In the present study, applying positive airway pressure methods (CPAP or IMV) during CPB was associated with better postoperative ABG measurements and (A-a) DO_2_.

## 1. Background

Respiratory complications after cardiac surgeries in patients who undergo CPB are one of the most important concerns of cardiac surgeons and anesthesiologists. These post-operative events may range from a minor respiratory impairment (46%) to acute respiratory distress syndrome (ARDS) (2%) (1) Atelectasis is one of the most common causes of these complications that can lead to hypoxia and pneumonia. Each of these complications increases the incidence of morbidity and mortality (2). The main inducing factors for atelectasis are; general anesthesia, CPB and discontinued blood perfusion of the lungs and gas exchange impairment during the surgery. Also opening the pleura may cause injuries to phrenic nerve. Harvesting mammary arteries in CABG cases may also result into similar condition, which is atelectasis. Continuous or progressive atelectasis can cause hypoxia and intra pulmonary shunt and therefore, post-operative respiratory impairment (3). The diagnosis of hypoxia is based on its related signs and symptoms, pulse oximetry and arterial blood gas (ABG) analysis. The treatment includes delivering of the oxygen that can be performed by different devices such as mask, nasal or mechanical ventilation. Different techniques and methods have been previously conducted to prevent these complications but there are still controversies over their benefits.

## 2. Objectives

In this study the efficacy of methods of positive airway pressure (continuous positive airway pressure) CPAP and intermittent mandatory ventilation; IMV) during CPB in patients undergoing CABG in prevention of post-operative complications have been compared.

## 3. Materials and Methods

This study protocol was granted by the Institutional Review Board; written informed consents were obtained from each patient, 300 patients over 18 years of age, with the mean age of 60.19 ± 9.5, candidates for elective CABG (on-pump) were enrolled in this prospective interventional trial from February to August 2011. The cases were randomly (balanced block randomization, block of six) divided into three groups: group A: CPAP patients, 102 patients received CPAP (Drager- Evita, Darger Medical, Inc; Germany) at 10 cmH2O during CPB, group B: IMV cases, 95 cases received IMV (Drager- Evita, Darger Medical, Inc; Germany) with tidal volume of 2cc/kg and respiratory rate 15/min. In control group C: 103 patients in received no types of ventilation during CPB. The patients excluded from the study were as follows: severe left main coronary artery disease, cases with the history of chronic obstructive pulmonary disease (COPD), renal and/or liver failure, as were the drug abusers. Arterial cannulation of a large peripheral vein was performed prior to inducing anesthesia to allow monitoring of the blood pressure. After three minutes of pre-oxygenation, for all the patients anesthesia was induced with the aid of Midazolam; 0.14 mg/kg, Fentanyl 10 μg/kg and Atracurium 0.5 mg/kg also they were ventilated with O_2 _100% for 3 minutes. After which intra-tracheal intubation was performed, a central vein catheter was inserted through the internal jugular vein. The infusion of Fentanyl 5-10 μg /kg/h, Midazolam 10 μg /kg /min and Atracurium5-10 μg /kg/min were continued in order to continue the anesthesia. All the anesthetic drugs were injected thorough central vein and before CPB, all the cases were mechanically ventilated with tidal volume of 10 cc/kg and respiratory rate 10/min and O_2_ 100%. Cerebral state monitor (CSM) and nerve stimulator were utilized to control the level of anesthesia and muscle relaxation. At the time of admission to the intensive care unit (ICU), all the patients were ventilated by synchronized intermittent mandatory ventilation (SIMV) mode with respiratory rate 10-12/min , tidal volume of 10ml/kg and FiO_2_ 60% ,and pressure controlled ventilation (PCV)10 cm H_2_O and PEEP 5cm H_2_O. The ABG analyses were controlled to maintain PCO_2_:35-40mmHg and PO_2 _> 70mmHg.All the cases received infusion of morphine 1mg/h with the method of patient controlled analgesia (PCA) in order to control the pain while in ICU. Based on the same protocol which includes, orientation, body temperature > 36 ºc, spontaneous respiratory function, pulmonary static compliance and ABG analyses with the results of PCO_2_ < 50mmHg, PO_2 _> 70mmHg and normal PH without active bleeding, all the patients were weaned off pain medication. After which the patients received O_2 _50% by venture mask. All the demographic data, CPB time, duration of ventilation, post-operative ABG analysis (PO_2_, PCO_2_, and O_2 _sat performed by ABG analyzer: Techno Medica Gastat 603I; USA), body temperature and respiratory rate were collected. In addition gas exchange index including, PaO_2_/FiO_2_, P (A-α) O_2_, the amount of respiratory dead space, pulmonary dynamic and static compliance were evaluated in eight different stages: before intratracheal intubation (O_2_ 21%) and prior to connecting the patients to CPB (O_2_ 50%). After connecting the patients to CPB ( O_2_ 100%), upon admission to ICU (O_2_ 60%),4,12,24 and 48 hours following ICU admission(O_2 _50%), chest xrays were performed at the time of ICU admission, 24 and 48 hours post-operative, standing orders for Post-operative atelectasis and pleural effusion were ordered by the radiologist based on the patient's chest X-rays.

### 3.1. Statistical Analysis

Statistical analyses were performed with SPSS 15 for Windows (SPSS Inc., Chicago, Illinois). Data were presented as mean+_standard deviation for interval and count (%) for categorical variables. Associations between ventilation method and interval data were investigated by one-way analysis of variance (ANOVA) models. Pair-wise comparisons performed by Bonferroni’s post-hoc test. Pearson’s chi square test was applied to find the associations between categorical factors and ventilation. Pattern of changes in the study outcomes and their relationships with study groups were assessed by repeated measure ANOVA models. , Chi square test and repeated measure ANOVA. A value of P < 0.05 was considered statistically significant.

## 4. Results

In this study 300 patient's candidate for elective CABG (on-pump)were enrolled and divided in three groups A,B,C: group A, received CPAP during CPB: 102 cases (34%),group B, received IMV during their surgery: 95 cases (31.7%) and control group; group C received no types of ventilation: 103 cases (34.3%). Among total, 202 patients were male (67.3%) and 98 patients were female (32.7%). The three study groups were matched well regarding pre-operative patients characteristics (Table 1). There were no significant differences regarding the intra-operative data, which includes: number of grafts and CPB time (min).However, the mean time of operation in group A was 227.01 ± 49.8(min) versus 282.16 ± 46.2 (min) in group B and 278.52 ± 52.8(min) in group C with P < 0.001 (Table 2). The results of ABG analysis which include PO_2_,PCO_2_,O_2_ saturation and also PaO2/FiO2, P (A-α) O2 were evaluated and compared in 8 different stages: before intratracheal intubation, before and after connecting the patients to CPB ,at the time of admission to the ICU,4,12,24 and48 hours after ICU admission. Prior to intratracheal intubation the mean value of PO2 was not significantly different between three groups: in-group A it was 62.99 ± 8.3mmHg versus group B: 64.12 ± 14.2mmHg and group C: 65.79 ± 11.7mmHg (P = 0.454). However, there were significant differences in mean value of PO2 between groups in the other seven evaluating times (P < 0.05). Table 3 demonstrates the differences in PO_2_ among the study groups. Table 4 demonstrates the results of PCO_2_ in all 8 different evaluating times. This variable was statistically different between the study groups before intubation, before and after CPB, 12 and 48 hours after extubation (P < 0.05). As for percentage of O_2_ saturation, the differences were significant before and after CPB, at the time of ICU admission and 12 hours after extubation (P < 0.05). In Table 5 the results of O_2_ sat and the differences between groups have been shown. The results of PaO2/FiO2 were also compared between groups and except for the first time of evaluation which was before intubation (group A: 299.95 ± 39.66 versus group B: 305.31 ± 67.87 versus group C: 341.14 ± 288.92, P = 0.401), this variable was significantly different at other evaluating times (P < 0.001). Table 6 shows the results and differences between the study groups regarding this variable. The same results have been reported for P(A-α) O2.Before intubation the mean value of P (A-α) O2 was 26.55 ± 8.93 in group A versus 25.98 ± 15.06 in group B versus 27.14 ± 12.86 in group C (P = 0.593). However, the results of this variable were statistically different in the other times (Table 7). The other factors, which were studied in our study, were the incidence of post-operative atelectasis and pleural effusion based on the reports of the chest x-rays which were performed at 3 different times: upon admission to the ICU, 24 and 48 hours post-operative. There were no significant differences between the study groups regarding the incidence of post-operative atelectasis and pleural effusion (Table 8).

**Table 1.** Baseline Characteristics of the Patients

Variable^a^	Study Group				P value
	CPAP^b^(n = 102)	IMV^b^(n = 95)	Control (n = 103)	Total (n = 300)	
**Gender**					0.446
Male	64 (62.7)	65 (68.4)	73 (70.9)	201 (67.3)	
Female	38 (37.3)	30 (31.6)	30 (29.1)	98 (32.7)	
**Age, y**	60.45 ± 9	60.75 ± 10.5	59.41 ± 9.5	60.19 ± 9.5	0.580
**DM** ^b^	80 (26.7)	30 (29.4)	22 (23.2)	28 (27.2)	0.605
**HTN** ^b^	13 (12.7)	23 (24.2)	33 (32.0)	69 (23.0)	0.004
**EF** ^b^	42.75 ± 11	44 ± 11.5	44.17 ± 10	43.63 ± 11	0.605

^a^ Values are n (%) or mean ± SD

^b^ Abbreviations: CPAP, continuous positive airway pressure; DM, diabetes mellitus; EF, ejection fraction; HTN, hypertension; IMV, intermittent mandatory ventilation

**Table 2.** Intra-operative Variables

Variable ^a^	Study Group				P value
	Total (n = 300)	CPAP^b^(n = 102)	IMV^b^(n = 95)	Control (n = 103)	
**Graft No**	3.04 ± 0.8	3.10 ± 0.8	2.92 ± 0.7	3.10 ± 0.8	0.178
**CPB** ^b^ **, min **	97.21 ± 34.5	97.44 ± 36.6	98.85 ± 34.7	95.47 ± 32.1	0.769
**Operation time, min**	262.16 ± 55.6	227.01 ± 49.8	282.16 ± 46.2	278.5 ± 52.8	< 0.001

^a^ Values are mean ± SD

^b^ Abbreviation: CPAP, continuous positive airway pressure; CPB, cardiopulmonary bypass; IMV, intermittent mandatory ventilation

**Table 3.** Partial Pressure of Arterial Oxygen, PO_2_ (mmHG)

Time	Study Group ^a^				P value
	Total (n = 300)	CPAP^b^(n = 102)	IMV^b^(n = 95)	Control (n = 103)	
**Before intubation**	64.31 ± 11.5	62.99 ± 8.3	64.12 ± 14.2	65.79 ± 11.7	0.454
**Before CPB^b^**	265.65 ± 180.9	228.72 ± 71.2	274.62 ± 303.2	234.54 ± 68.0	< 0.001
**After CPB**	195.36 ± 83.0	226.53 ± 86.5	197.16 ± 78.0	162.83 ± 71.6	< 0.001
**ICU admission**	117.42 ± 44.8	130.82 ± 51.3	116.14 ± 36.2	105.32 ± 41.6	< 0.001
**4 hours afterextubation**	94.45 ± 20.65	98.13 ± 17.5	96.09 ± 16.8	89.30 ± 25.4	< 0.001
**12 hours afterextubation**	89.04 ± 19.5	93.17 ± 12.6	92.96 ± 19.1	81.33 ± 23.1	< 0.001
**24 hours afterextubation**	86.28 ± 15.7	91.76 ± 11.8	88.39 ± 13.1	78.89 ± 18.4	< 0.001
**48 hours afterextubation**	85.93 ± 15.4	90.24 ± 9.7	87.97 ± 13.6	79.79 ± 19.3	< 0.001

^a^ Values are mean ± SD

^b^ Abbreviations: CPAP, continuous positive airway pressure; CPB, cardiopulmonary bypass; IMV, intermittent mandatory ventilation

**Table 4.** Partial Pressure of Arterial Carbon Dioxide; PCO_2_ (mmHg)

Time	Study Group ^a^				P value
	Total (n = 300)	CPAP^b^(n = 102)	IMV^b^(n = 95)	Control (n = 103)	
**Before intubation**	32.06 ± 4.5	33.04 ± 4.3	32.86 ± 4.2	30.34 ± 4.3	< 0.001
**Before CPB^b^**	29.9 ± 6.04	30.85 ± 5.8	30.31 ± 6.8	28.62 ± 5.3	0.022
**After CPB**	33.6 ± 6.3	34.93 ± 6.8	33.61 ± 6.3	32.23 ± 5.5	0.037
**ICU admission**	33.6 ± 6.3	34.23 ± 7.0	33.84 ± 5.4	32.70 ± 6.2	0.198
**4 hours afterextubation**	34.9 ± 4.6	35.55 ± 4.7	34.78 ± 4.9	34.28 ± 4.0	0.103
**12 hours afterextubation**	34.9 ± 4.5	36.06 ± 4.6	34.11 ± 4.0	34.46 ± 4.6	0.001
**24 hours afterextubation**	34.72 ± 4.4	35.43 ± 4.2	34.68 ± 4.5	34.05 ± 4.4	0.079
**48 hours afterextubation**	34.5 ± 5.1	35.87 ± 6.3	34.46 ± 4.2	33.34 ± 4.2	0.002

^a^ Values are mean ± SD

^b^ Abbreviations :CPAP, continuous positive airway pressure; CPB, cardiopulmonary bypass; IMV, intermittent mandatory ventilation

**Table 5.** The Percentage of Oxygen Saturation; O_2_ sat (%)

Time	Study Group ^a^				P value
	Total (n = 300)	CPAP^b^(n = 102)	IMV^b^(n = 95)	Control ( n = 103)	
**Before intubation**	92.12 ± 3.6	92.14 ± 3.2	91.46 ± 4.6	92.70 ± 2.8	0.281
**Before CPB^b^**	99.96 ± 5.8	99.81 ± 0.4	100.59 ± 1 0.3	99.53 ± 0.7	0.003
**After CPB**	98.11 ± 5.0	99.09 ± 1.0	98.55 ± 2.2	96.75 ± 8.1	< 0.001
**ICU admission**	99.22 ± 52.1	96.90 ± 3.2	105.96 ± 92.4	95.30 ± 4.4	0.003
**4 hours afterextubation**	95.74 ± 2.8	95.99 ± 2.3	96.25 ± 1.2	95.01 ± 3.6	0.113
**12 hours afterextubation**	95.06 ± 2.8	95.44 ± 2.3	95.26 ± 2.7	94.50 ± 3.3	0.079
**24 hours afterextubation**	95.00 ± 2.8	95.24 ± 2.4	95.31 ± 2.2	94.49 ± 3.5	0.380
**48 hours afterextubation**	94.87 ± 3.2	95.23 ± 2.1	95.26 ± 2.2	94.16 ± 4.5	0.448

^a^ Values are mean ± SD

^b^ Abbreviations: CPAP, continuous positive airway pressure; CPB, cardiopulmonary bypass; IMV, intermittent mandatory ventilation

**Table 6.** The Ratio of Partial Pressure of Arterial Oxygen to the Fraction of Inspired Oxygen, PO_2_/FiO_2_ (mmHg)

Time	Study Group ^a^				P value
	Total (n = 300)	CPAP^b^( n = 102)	IMV^b^( n = 95)	Control (n = 103)	
**Before intubation**	315.79 ± 175.5	299.95 ± 39.66	305.31 ± 67.87	341.14 ± 288.92	0.401
**Before CPB^b^**	442.75 ± 301.51	481.19 ± 118.78	457.70 ± 505.36	390.90 ± 113.43	< 0.001
**After CPB**	325.59 ± 138.3	377.54 ± 144.22	328.9 ± 130.04	271.37 ± 119.34	< 0.001
**ICU admission**	293.54 ± 112.04	327.05 ± 128.38	290.34 ± 90.70	263.30 ± 104.10	< 0.001
**4 hours afterextubation**	314.84 ± 68.86	327.09 ± 58.17	320.31 ± 55.96	297.66 ± 84.78	< 0.001
**12 hours afterextubation**	296.79 ± 65.13	310.55 ± 41.93	309.86 ± 63.68	271.10 ± 77.07	< 0.001
**24 hours afterextubation**	287.59 ± 52.47	305.88 ± 39.54	294.63 ± 43.67	262.97 ± 61.43	< 0.001
**48 hours afterextubation**	286.43 ± 51.38	300.78 ± 32.29	293.22 ± 45.34	265.95 ± 64.33	< 0.001

^a^ Values are mean ± SD

^b^ Abbreviations: CPAP, continuous positive airway pressure; CPB, cardiopulmonary bypass; IMV, intermittent mandatory ventilation

**Table 7.** The alveolar- arterial Oxygen Gradient, P (A-α) O_2_ (mmHg)

Time	Study Group ^a^				P value
	Total (n = 300)	CPAP^b^(n = 102)	IMV^b^(n = 95)	Control (n = 103)	
**Before intubation**	26.58 ± 12.45	26.55 ± 8.93	25.98 ± 15.06	27.14 ± 12.86	0.593
**Before CPB^b^**	80.33 ± 74.87	46.47 ± 71.2	90.74 ± 70.8	104.28 ± 70.43	0.022
**After CPB**	134.81 ± 83.15	103.60 ± 86.38	131.77 ± 77.16	168.51 ± 72.55	< 0.001
**ICU admission**	90.14 ± 45.86	75.88 ± 52.02	90.90 ± 36.99	103.54 ± 42.89	< 0.001
**4 hours afterextubation**	49.18 ± 21.50	44.95 ± 19.23	47.35 ± 18.2	55.08 ± 25.05	0.002
**12 hours afterextubation**	54.35 ± 20.88	48.66 ± 4.22	51.30 ± 19.83	62.80 ± 24.55	< 0.001
**24 hours afterextubation**	57.16 ± 17.8	50.81 ± 14.18	55.07 ± 14.99	65.36 ± 20.44	< 0.001
**48 hours afterextubation**	57.95 ± 17.47	52.49 ± 12.13	55.70 ± 15.21	65.45 ± 21.08	< 0.001

^a^ Values are mean ± SD

^b^ Abbreviations: CPAP, continuous positive airway pressure; CPB, cardiopulmonary bypass; IMV, intermittent mandatory ventilation

**Table 8.** The Incidence of Post-operative Atelectasis and Pleural Effusion

Variable ^a^	Study Group				P value
	Total (n = 300)	CPAP^b^(n = 102)	IMV^b^(n = 95)	Control ( n = 103)	
**Atelectasis**					
ICU admission	71 (23.7)	28(27.5)	26 (27.4)	17 (16.5)	0.108
24 hours after surgery	91 (30.3)	29 (28.4)	26 (27.4)	36 (35 )	0.447
48 hours after surgery	91 (30.3)	29 (28.4)	26 (27.4)	36 (35)	0.447
**Pleural effusion**					
3 (3.2)	7 (2.3)	3 (2.9)	3 (3.2)	1 (1)	0.525
3 (3.2)	11 (3.7)	3 (2.9)	3 (3.2)	5 (4.9)	0.729
3 (3.2)	11 (3.7)	3 (2.9)	3 (3.2)	5 (4.9)	0.729

^a^ Values are n (%)

^b^ Abbreviations: CPAP, continuous positive airway pressure; IMV, intermittent mandatory ventilation

## 5. Discussion

Open-heart surgeries and CPB alter the pulmonary function and cause multiple complications that varied from microscopic atelectasis to acute respiratory distress syndrome. Post-operative atelectasis is a common complication that different factors are responsible for its incidence (4). In an anesthetized patient, the lung tissue is compressed due to diaphragm relaxation and its upside movement (5). Also during CPB, the occurrence of atelectasis is common as a result of increased pleural space pressure and collapsed lungs with no perfusion (6). In addition, the activation of inflammatory mediators in systemic inflammatory response during open-heart procedures results into atelectasis and gas exchange impairment. Pre-oxygenation and induction of anesthesia with O_2_ without positive pressure ventilation might also induce atelectasis. Different studies have been conducted before in order to evaluate the effect of CPB on post-operative pulmonary complications. In a study by Magnusson et al. (7), they evaluated the correlation of atelectasis and CPB. Eighteen pigs divided in three groups, six pigs received standard CPB (bypass group), six other pigs had the same procedure without CPB and another six pigs were anesthetized for the same duration without any surgery. According to the results, they concluded that atelectasis is produced to a much larger extent after CPB than after anesthesia alone or with the sternotomy and it explains most of the post-CPB increase in shunt and hypoxia. The aim of this study was to evaluate the effect of mechanical ventilation on the incidence of atelectasis in patients undergoing on-pump CABG. Among the three study groups (group A: CPAP group, group B: IMV group and group C: Control group) in our trial there were no significant differences regarding the incidence of post-operative atelectasis and pleural effusion based on radiographic data. However, impossibility to perform CT scans and MRIs in order to detect atelectasis for all the patients were the study limitations of this research. Another variable, which was compared in our study groups, was the mean value of PO_2_ that was not statistically different between groups before intratracheal intubation. However, at the other seven evaluating times this variable was significantly higher in both CPAP and IMV group compared to the control group. The same results have been achieved for PO_2_/FiO_2_. As for P (A-α) O_2_, our study also revealed that the mean value of P (A-α) O_2_ was significantly lower in CPAP and IMV group than control group. The same results have been demonstrated in a study of Magnusson et al. (8), in which 14 patients candidate for elective CABG were divided in two groups .Seven patients received CPAP 10cm H_2_O and the other seven cases underwent CABG with no intervention. ABG analyses were studied before CPB, after CPB and 4 hours after the surgery. They concluded that with the aid of CPAP 10 cm H_2_O, gas exchange index such as PaO2, P (A-α) O_2_ were significantly improved. In another study by Koner et al. (9), P (A-α) DO_2_ was evaluated in 44 patients undergoing CABG and CPB. The patients were divided in three groups. First group: 15 patients ventilated with protective tidal volumes 6 ml/kg and PEEP 5 cm H_2_O, second group; 14 patients received mechanical ventilation with conventional tidal volume 10 ml/kg and PEEP 5 cm H_2_O and third group; 15 patients ventilated with conventional tidal volumes without PEEP (CV + ZEEP). As a result they observed that oxygenation and alveolar-arterial oxygen difference were better in both PEEP groups than CV + ZEEP group 24 h after the surgery. The results of this investigation indicate that applying positive airway pressure methods (CPAP or IMV ) during CPB was associated with better post-operative ABG measurements and (A-a) DO_2_ and may also help attenuate post-operative pulmonary complications ,commonly observed in patients after exposure to cardiopulmonary bypass.
